# SQSTM1/p62 Promotes Cell Growth and Triggers Autophagy in Papillary Thyroid Cancer by Regulating the AKT/AMPK/mTOR Signaling Pathway

**DOI:** 10.3389/fonc.2021.638701

**Published:** 2021-04-15

**Authors:** Fangqin Yu, Runsheng Ma, Chenguang Liu, Lele Zhang, Kaixiang Feng, Meiqi Wang, Detao Yin

**Affiliations:** Department of Thyroid Surgery, The First Affiliated Hospital of Zhengzhou University, Zhengzhou, China

**Keywords:** SQSTM1/p62, papillary thyroid cancer, proliferation, autophagy, TPC-1

## Abstract

**Background:**

Thyroid cancer is one of the most common endocrine malignancies worldwide, and papillary thyroid cancer (PTC) is the most common pathologic type of thyroid cancer. SQSTM1/p62 activity mediates different biological functions. This study aimed to investigate the effect of SQSTM1/p62, a multifunctional receptor, on biological function and autophagy characteristics in the human PTC cell line TPC-1.

**Methods:**

A total of 105 primary PTC samples and matched adjacent normal thyroid tissue samples were obtained to evaluate the expression of p62 in clinical patients. A similar p62 expression pattern was found in PTC cell lines and normal human thyroid follicular epithelial cells. To evaluate the effect of SQSTM1/p62 on TPC-1 cells, we constructed the p62 knockout cell line p62-KO-TPC-1. Cell proliferation, cell cycle, and cell apoptosis were analyzed by colony formation tests, Cell Counting Kit-8 (CCK-8) assays and flow cytometry *in vitro*. TPC-1 and p62-KO-TPC-1 human PTC cell lines in the logarithmic growth phase were subcutaneously implanted into BALB/c nude mice to verify their proliferation effect *in vivo*. Furthermore, western blotting and immunohistochemistry (IHC) were used to detect the expression of AKT/AMPK/mTOR signaling pathway-related proteins.

**Results:**

Overall, p62 expression was higher in tumor tissues than in normal tissues in 73 of 105 PTC patients (69.5%). The expression level of p62 in the PTC cell line was higher than that in the normal thyroid cell line. Our data indicated that *in vitro*, p62 deficiency could decrease the number of colonies, inhibit cell growth and the cell cycle, and induce apoptosis. Tumor xenograft experiments in BALB/c nude mice corroborated these findings. Moreover, the molecular mechanism was explored by western blotting, and we found that the AMPK/AKT/mTOR pathway was involved.

**Conclusions:**

The results indicate that p62 might mediate cell autophagy and apoptosis in TPC-1 cells *via* the AMPK/AKT/mTOR pathway and could be used as a potential therapeutic approach for PTC.

## Introduction

Epithelial follicular-cell-derived thyroid cancer is one of the most common malignancies of the endocrine system worldwide, and its incidence has sharply in recent years (1–7). Internationally, thyroid cancer is the most common cancer in women in Korea (8) and the fifth most common cancer in women in the USA (9). Papillary thyroid carcinoma (PTC) is the most common pathologic type of thyroid cancer, accounting for more than 80% according to reports (10–12). Although differentiated thyroid cancer, including PTC and follicular thyroid cancer, is associated with low mortality, the disease recurrence rate is high, at 20-30%, or even higher in some subgroups of patients (13). Despite the high incidence and prevalence of PTC, most patients have a good prognosis after standard treatment, which is surgery followed by radioiodine and regular reexamination. However, inoperable and radioiodine-refractory PTC may result in death, and there is no effective treatment solution (14).

The occurrence and development of thyroid tumors is a complex process involving multiple genes, including cancer suppressor genes, cancer promoting genes and many regulator genes (15, 16). It is widely believed that autophagy and metabolic systems help tumor cells survive by circulating the distribution of proteins within cells. In our previous study, we found that p62 was upregulated in PTC tissue compared to normal thyroid tissue by PCR. Therefore, we assumed that p62 occupied an important position in PTC and made multiple accurate internal homeostasis regulating the growth of PTC cells.

The sequestosome 1 gene (SQSTM1), also named p62, is located in 5q35, and it is an adaptor protein in the ubiquitination system as well as a cargo protein receptor in selective autophagy that plays an important role in regulating intracellular protein degradation (17). Nevertheless, it was reported that p62 also participates in many cellular biological activities, such as the cell cycle, cell metabolism (18), the scavenging effect of selenium on peroxy radicals, etc. Mutations in the p62 gene are strongly associated with Paget’s disease of the bone (19), murine myeloid leukemia progression (20), neurodegenerative disease (21), obesity (22), vascular senescence (23), aging pathologies and cancer (24, 25). p62 consists of 440 amino acids covering more than 10 domains and binding sites; hence, it is a key center of regulating multiple activities of cells, such as insulin signaling, energy balance, adipogenesis, brown adipose tissue (BAT) thermogenesis, inflammation, oxidative stress, apoptosis, etc (26–30).

An increasing volume of evidence indicates that p62 participates in many signal transduction pathways, including insulin, Keap1-Nrf2, ERK, and p38/MAPK signaling pathways (31). Therefore, we discussed the effects of p62 on the physiology and function of PTC cells from multiple aspects and further studied the pathogenesis of PTC.

## Materials and Methods

### Patients

A total of 105 primary PTC samples and matched adjacent normal thyroid tissue samples were obtained at the time of initial surgery and snap-frozen immediately after tumor removal. Inclusion criteria: all patients had not received radiotherapy, chemotherapy or other treatment measures before surgery; all patients underwent thyroid cancer surgery for the first time; radical operation of thyroid carcinoma was performed in all cases; pathological results were double-blind review by pathologists and diagnosed as papillary thyroid carcinoma. Exclusion criteria: the patients had a history of other malignant tumors; accompanied by cardiac insufficiency, liver and kidney failure and other important organs of serious lesions; malignant infection.

The study was approved by the Ethics Committee of Zhengzhou University, and human tissues were obtained with informed written consent from the patients. All tissue samples were reviewed by an endocrine pathologist to confirm the diagnosis. PTC samples estimated to contain more than 80% tumor cells were used. This study was approved by the ethical standards of the institutional ethics committee, and informed consent was obtained from all patients (No. 2019-KY-314).

From March 2019 to September 2019, 105 patients with pathologically proven PTC were included in this study. All patients were aged 24 to 62 years (mean age, 42.3 years).

### Cell Culture and Treatments

The human PTC cell line TPC-1 was kindly provided by Dr Ye Lei (Shanghai Rui Jin Hospital, Shanghai, China) and the normal human thyroid follicular epithelial cell line Nthy-ori3-1 was purchased from Shanghai Cell Biochemical Institute (Shanghai, China). All the cell lines were cultured in RPMI-1640 medium (Solarbio, Beijing, China) with 10% fetal bovine serum (FBS) (Gemini, USA) and incubated in a humidified atmosphere of 5% CO_2_ at 37°C. The following chemicals were used: rapamycin (Dalian Meilun Biotechnology: XB13514, 10nM) and bafilomycin A1 (Solarbio: A8510, 5µM and 10µM).

### CRISPR/Cas9

To investigate the effect of p62 on TPC-1 cells, the CRISPR/Cas9 technique was used to construct the p62 knockout cell model p62-KO-TPC-1. The p62 knockout colony was generated by CRISPR/Cas9-mediated genome editing. The sgRNA was designed and synthesized to specifically target p62. The sgRNA guide sequence was 5′-TGGCTCCGGAAGGTGAAACA-3′. The synthesized and validated Lenti-CAS9-sgRNA-Puromycin vector was transferred into TPC-1 cells. Single cells were expanded to obtain individual clones that were lysed and quantified. The absence of p62 was verified by western blotting and RT-qPCR.

### CCK-8 Cell Proliferation and Colony Formation Assays

A Cell Counting Kit-8 assay (Dojindo, Japan) was first performed. Tumor cells were cultured in 96-well plates at a density of 1000 cells per well. The next day, 10 μl CCK-8 reagent was added to each well. After a further incubation of 4 hours at 37°C, the cell proliferation rate was assessed by measuring the absorbance at 450 nm with the Universal Microplate Reader.

Colony formation assays were subsequently conducted. A total of 500 cells were plated in 6-cm dishes for 2 weeks. Then, the cells were fixed with methanol for 20 minutes and stained with crystal violet. The colonies were counted.

Data are shown from three independent experiments performed in triplicate wells.

### Xenograft Assay in Nude Mice

A total of 2×10^6^ cells were collected and injected subcutaneously into the flank regions of 4-week-old female BALB/c nude mice (Vital River, Beijing, China). The tumor volume was measured at the indicated time after inoculation. After 3 weeks, the tumor-bearing mice were sacrificed, and the average weight of the tumor tissues was measured. Sections of tumor tissues were detected by immunohistochemistry (IHC). The study was approved by the Ethics Committee of the First Affiliated Hospital of Zhengzhou University (No. 2019-KY-314).

### Quantitative Real-Time PCR

The expression of the p62 gene was detected by RT-qPCR. The primers for the p62 and GAPDH genes were synthesized by Shanghai Sangon Biotech, and the sequences were as follows: p62 forward, 5’-AGGCGCACTACCGCGAT-3’ and reverse, 5’-CGTCACTGGAAAAGGCAACC-3’; GAPDH forward, 5’-GGTCGTATTGGGCGCCTGGTC-3’ and reverse, 5’-TGACGGTGCCATGGAATTTGCCA-3’. Total RNA was extracted from primary PTC samples and matched adjacent normal thyroid tissue samples, TPC-1 and p62-KO-TPC-1 cells (continuously cultured in puromycin-free medium for ≥4 weeks) using TRIzol^®^ reagent (Invitrogen; Thermo Fisher Scientific, Inc.), and the PrimeScript RT reagent Kit with gDNA Eraser (TaKaRa, Osaka, Japan) was used to remove genomic DNA and synthesize complementary DNA (cDNA). Quantitative real-time PCR was performed in triplicate using the RT-qPCR mRNA SYBR Green Detection kit (TaKaRa, Osaka, Japan). The threshold cycle (Ct) value was recorded, and changes in mRNA levels were determined by the 2^-ΔΔCt^ method using GAPDH for internal crossing normalization.

### Apoptosis Detection

The Annexin V-FITC Apoptosis Detection Kit (KeyGen BioTECH: KGA105-KGA108) was used to assay the apoptosis of TPC-1 and p62-KO-TPC-1 cell lines. The apoptotic cells labeled with annexin V were assessed by a flow cytometer (Beckman, CA). Data were obtained from three independent experiments.

### Cell Cycle Analysis

The Cell Cycle Detection Kit (KeyGen BioTECH: KGA511-KGA512) was used to assay the cell cycle of TPC-1 and p62-KO-TPC-1 cells. The tumor cells labeled with PI/RNase A fluid were detected by a flow cytometer (Beckman, CA), and the data were analyzed using Multicycle-DNA Cell Cycle Analysed Software. Data were obtained from three independent experiments.

### Western Blotting Analysis

After culturing in RPMI-1640 medium with 10% FBS, TPC-1 and p62-KO-TPC-1 cells were lysed in R IPA lysis buffer (Cwbiotech, China) containing 1% Protease Inhibitor Cocktail (Cwbiotech, China). Equal amounts of protein were separated by sodium dodecyl sulfate polyacrylamide gel electrophoresis (SDS-PAGE) and transferred to a polyvinylidene fluoride (PVDF) membrane (Merck Millipore, Germany). After blocking with 5% skimmed milk, the PVDF membrane was then incubated with the following antibodies: p62 (Abcam: ab109012, 1/20000), AKT (Servicebio Technology: GB111114, 1/1000), P-AKT (Affinity Biosciences: AF0908, 1/1000), AMPK (Abcam: ab32112, 1/2000), Bax (Abcam: ab32503, 1/2000), Bcl-2 (Abcam: ab32124, 1/1000), mTOR (Cell Signaling Technology: 2983, 1/1000), LC3A (Sigma Aldrich: L8793, 1/1000), actin (Servicebio Technology: GB12001, 1/1000), and GAPDH (GOOD HERE: AB-P-R 001, 1/1000).

### Statistical Analysis

The results are expressed as the mean ± SD for at least three separate experiments. Statistical evaluation was performed using Prism (GraphPad) software. Statistical significance was assessed using a two-tailed unpaired Student’s t-test, Chi-square test and Fisher’s exact test. P < 0.05 was considered to indicate a statistically significant result.

## Results

### The Expression of p62 Is Increased in Clinical PTC Tissues and Cell Lines Compared to Controls and Is Associated With Tumor Size

**Figure 1:**
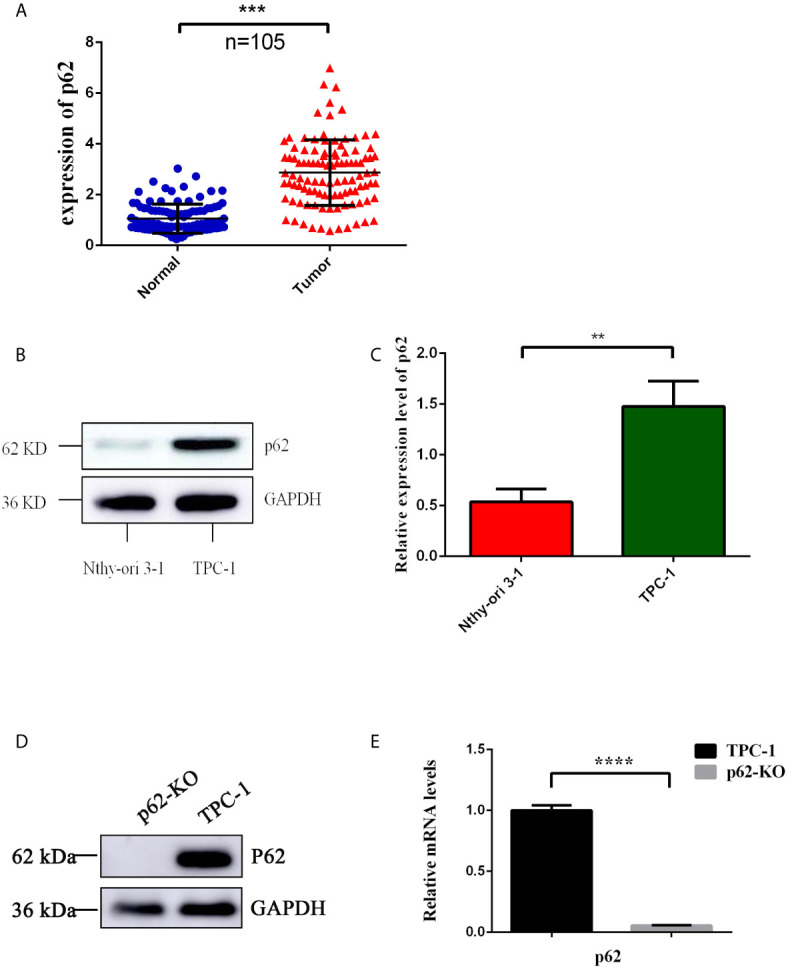
Expression of p62 in PTC tissues and cells. **(A)** Up-regulated expression of p62 was observed in PTC tissues. **(B)** Up-regulated expression of p62 was observed in PTC cells by WB. **(C)** Up-regulated expression of p62 was observed in PTC cells by RT-qPCR. **(D)** Knock-out efficiency verified by WB. **(E)** Knock-out efficiency verified by RT-qPCR. (**P < 0.01, ***P < 0.001, ****P < 0.0001).

To examine the expression of p62 in PTC, we assessed p62 expression by RT-qPCR in 105 PTC samples and their matched normal thyroid tissues. Out of 105 paired samples, 73 presented higher expression of p62 in tumor tissues than in normal tissues (69.5%). The results showed that the p62 expression level was significantly increased in PTC tissues compared to normal thyroid tissues. As shown in **Figure 1A**, the levels of p62 were frequently upregulated in PTCs. To further investigate the clinicopathological and prognostic significance of p62 levels in PTC patients, the p62 expression level was dichotomized into two groups, positive and negative, and we found that the expression of p62 was correlated with tumor size (*p*=0.0066) (**Table 1**).

**Table 1:** The relationship between p62 expression and clinicopathological features in PTC.

Clinical data	Expression of p62		p
	Positive	Negative	
**Sex**			
male	20.0%(21/30)	8.6%(9/30)	0.9465
female	49.5% (52/75)	21.9%(23/75)	
**Age**			
<55	58.1% (61/87)	24.8% (26/87)	0.7832
≥55	11.4% (12/18)	5.7% (6/18)	
**Tumor size**			
<2cm	61.0% (64/84)	19.0% (20/84)	0.0066
≥2cm	8.6% (9/21)	11.4% (12/21)	
**TNM stage**			
I	56.2% (59/81)	21.0% (22/81)	0.2096
II~IV	13.3% (14/24)	9.5% (10/24)	
**Lymph node metastasis**			
NO	28.6% (30/48)	17.1% (18/48)	0.2019
YES	41.0% (43/57)	13.3% (14/57)	
**			

TNM, Tumor Node Metastases.

Then, we detected p62 expression in the PTC cell line TPC-1 *in vitro* and in a normal human thyroid follicular epithelial cell line (Nthy-ori3-1). Similar to the results obtained using tissue samples, p62 expression was higher in the PTC cell line than in normal human thyroid follicular epithelial cells (**Figures 1B, C**). Collectively, these results revealed that p62 is markedly upregulated in PTC tissues and cell lines, suggesting that it might play a tumor promoter role in PTC.

### p62 Deficiency Inhibits the Proliferation of TPC-1

To investigate the effect of p62 on TPC-1 cells, the CRISPR/Cas9 technique was used to construct the p62 knockout cell model p62-KO-TPC-1. To verify the knockout efficiency, western blotting and RT-qPCR were used to detect the protein and mRNA expression of p62 (**Figures 1D, E**).

**Figure 2:**
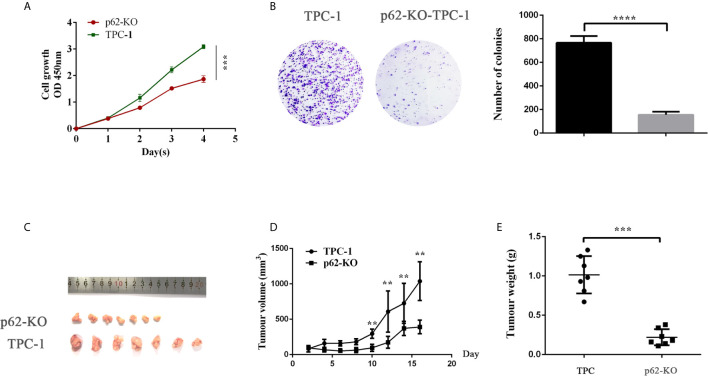
p62 deficiency decreases TPC-1 cell proliferation in vitro and in vivo. **(A)** The proliferation of papillary thyroid cancer TPC-1 and p62-KO-TPC-1 cells was detected using a CCK-8 assay. **(B)** Cell colony formation assay showed cell viability after p62 was knockout. **(C)** At day 16 after injection, smaller tumors were formed after p62 knockout. The volumes **(D)** and weights **(E)** of tumors were presented as mean ± SD (**P < 0.01, ***P < 0.001, ****P < 0.0001).

CCK-8 and colony formation assays were used to examine the effect of p62 on TPC cell proliferation. As shown in **Figure 4**, knocking out p62 cells suppressed the cell growth rate (**Figure 2A**) and colony formation (**Figure 2b**). Next, we tested whether p62 could influence the growth of TPC xenografts in nude mice. We established a xenograft model by subcutaneous injection of p62-KO TPC-1 cells into the flanks of nude mice. The results showed that the tumor growth rate and tumor weights in the p62-KO group were smaller than those of the control group (**Figures 2C–E**). Overall, these results indicated that p62 promotes proliferation.

### p62 Deficiency Induces the Cell Cycle and Apoptosis of TPC-1 Cells

**Figure 3:**
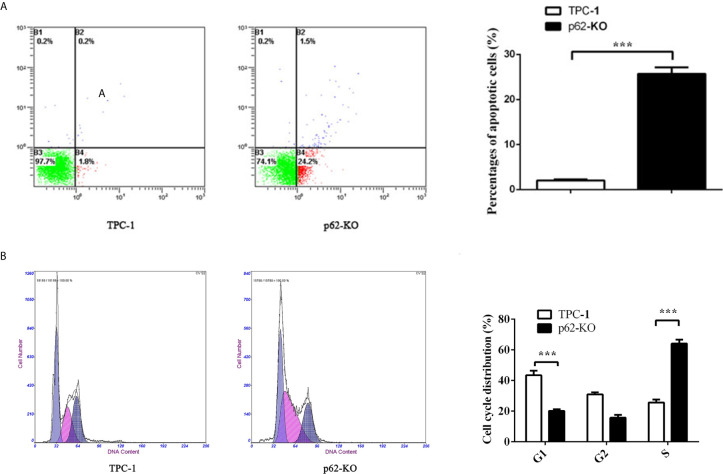
p62 deficiency increases cell apoptosis rate and block cell cycle in TPC-1 cells. **(A)** Representative photos (left) of flow cytometry assay and statistical plots (right) of cell apoptosis in TPC-1 and p62-KO-TPC-1 cells. **(B)** Representative photos (left) of flow cytometry assay and statistical plots (right) of cell cycle in TPC-1 and p62-KO-TPC-1 cells (***P < 0.001).

**Figure 4:**
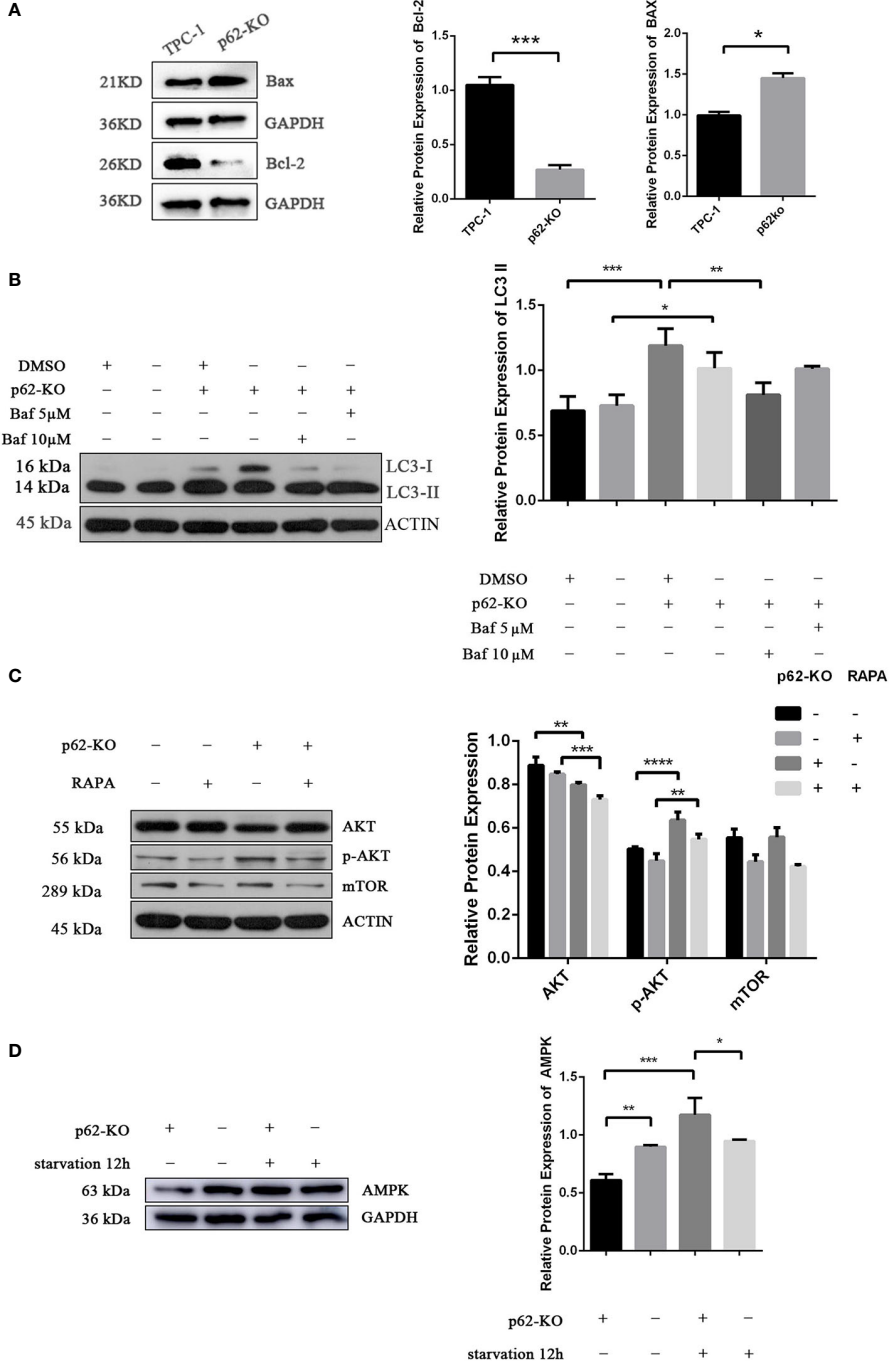
p62 deficiency mediates the level of AKT/ AMPK/mTOR related pathway. **(A)** Western blotting analysis the expression of Bax and Bcl-2 in TPC-1 and p62-KO-TPC-1 cells. **(B)** Western blotting showing the expression of LC3 in groups of DMSO, p62-KO, Baf (Bafilomycin A1) 5µM and Baf 10µM treatment of 12 hours in TPC-1 cells. **(C)** Western blotting analysis the expression of AKT and mTOR in p62-KO and RAPA (Rapamycin) treatment groups in TPC-1 cells. **(D)** Western blotting results showing the level of AMPK in p62-KO and starvation of 12 hours condition (*P < 0.05, **P < 0.01, ***P < 0.001, ****P < 0.0001).

To verify the impact of p62 expression on TPC-1 cell proliferation, we assessed cell cycle distribution. Flow cytometry analysis showed that inhibiting p62 in TPC-1 cells increased the percentage of cells in G1 phase and reduced the percentage of cells in S phase compared with that in the control (**Figure 3B**) which revealed that p62 deficiency blocked the cell cycle of TPC-1 at S phase. Then, we assessed the effect of p62 on cell apoptosis. The results using annexin V/PI double staining and flow cytometry in TPC-1 cells indicated that the number of apoptotic cells was increased when p62 was knocked out (**Figure 3A**). The expression of the apoptosis-related proteins Bax and Bcl-2 also verified that p62 deficiency induced apoptosis in response to TPC-1 (**Figure 4A**). Therefore, these results indicated that p62 affects cell proliferation by regulating the TPC-1 cell cycle and apoptosis.

### p62 May Affect Tumor Autophagy Through the AMPK/AKT/mTOR Pathway

To assess the impact of p62 on autophagy of TPC-1 cell line, the expression of the autophagy-related protein LC3 was detected by western blotting in TPC-1 and p62-KO-TPC-1 cells. As shown in **Figure 4B**, the expression of LC3-II was increased after p62 was knocked out in TPC-1 cells, but not significant accumulation was observed after Bafilomycin A1, a well-known inhibitor of autophagosome-lysosome fusion, administration at the dose of 5µM and 10µM for 12h. This data suggest that the increase in LC3-II levels observed after p62-knockout occurred due to inhibition of autophagy degradation and not to an increase in the autophagic flow.

Recent data reported that autophagy can be regulated through the AKT/AMPK/mTOR pathway (36).Thus, we verified if p62 triggers autophagy in thyroid cancer through this pathway, analysing the expression of related proteins. We found that the expression of AMPK and AKT was significantly decreased after p62 knockout (**Figures 4C, D**). However, p-AKT was increased in p62-KO-TPC-1 cells, which meant that p62 deficiency promoted the phosphorylation of AKT and activated its function. The expression of AKT was decreased and the expression of p-AKT was increased after treatment with rapamycin, an mTOR inhibitor, for 12h, in p62-KO-TPC-1 cells (**Figure 4C**), which meant that mTOR was involved in this process. However, there was no significant change in mTOR expression in p62-KO-TPC-1 cells, we speculated that there existed some nagetive feedback activation, which could be further investigated.

## Discussion

p62 is a cargo protein of ubiquitination substrates, and its LIR domain interaction with LC3 assists in complete protein degradation of specific substrates *via* transfer of the p62-protein complex to autophagolysosomes (32). Ubiquitylation of p62 suppresses dimerization of the UBA domain, liberating its ability to recognize polyubiquitylated substrates for selective autophagy (33). In-depth research has been performed, and the expression of p62 has even become a reliable indicator to measure autophagic flux. In this study, we first demonstrated that p62 is involved in the development of PTC by promoting cell growth and inhibiting cell apoptosis and autophagy. We found that p62 expression is higher in clinical PTC tissues and cell lines than in normal tissues and cells. Furthermore, p62 knockout inhibited cell growth and autophagy in the PTC cell line and further repressed tumor growth *in vivo*. Thus, our results provide evidence that p62 has a tumor-promoting effect in PTC.

AMPK-activated protein kinase (AMPK) is known as an energy sensor that regulates cellular metabolism by mediating the insulin pathway, and its phosphorylation is triggered by glucose stress or hypoxia in the tumor microenvironment (34). However, evidence shows that AMPK plays an important role in autophagy, age-related changes and many other molecular mechanisms (35). Recent studies revealed that autophagy can be regulated *via* the AKT/AMPK/mTOR pathway (36), while AKT, mTOR and AMPK play an important role in initiating and inducing autophagy (37). Considering that p62 deficiency inhibited autophagy in PTC, we hypothesized that the AKT/AMPK/mTOR signaling pathways may be involved in the autophagy induced by p62 in PTC. In support of this hypothesis, we found that p62 activated AKT/AMPK/mTOR signaling in PTC. Thus, our results suggested that p62 plays a role in autophagy by regulating the AKT/AMPK/mTOR signaling pathways in PTC.

In summary, LC3-II expression was increased after p62 knockout, but no significant accumulation was observed after Bafilomycin A1 application, suggesting that autophagy was inhibited. The western blotting results of related pathway proteins showed that p62KO promoted the phosphorylation of AKT, inhibited the expression of AMPK and caused the activation of mTOR, which made it difficult for autophagy to be started and led to autophagy inhibition, indicating that p62 played a role in inducing autophagy in papillary thyroid carcinoma. Enhancement of autophagy leads to enhanced decomposition of intracellular metabolic wastes, and the protein raw materials produced can generate proteins required by cells through a series of synthesis effects, maintain intracellular homeostasis, and help tumor cells to continue to survive under harsh conditions (38). Therefore, excessive accumulation of p62 in tumor cells is characterized by cell cycle initiation, inhibition of apoptosis, and thus enhanced proliferation ability; in animals, the tumor growth rate increased and the tumor volume increased; in clinical studies, the expression of p62 was positively correlated with tumor size. The specific mechanism involved still needs further study.

This study revealed the tumor-promoting effect of p62 in PTC by AKT/AMPK/mTOR pathway, contributing to the basic research on the occurrence and development of PTC, and further suggesting that p62 or p62 related molecule may be a diagnostic and therapeutic target for PTC.

## Data Availability Statement

The original contributions presented in the study are included in the article/supplementary material. Further inquiries can be directed to the corresponding author.

## Ethics Statement

The studies involving human participants were reviewed and approved by the Ethics Committee of the First Affiliated Hospital of Zhengzhou University. The patients/participants provided their written informed consent to participate in this study. The animal study was reviewed and approved by the Ethics Committee of the First Affiliated Hospital of Zhengzhou University.

## Author Contributions

All authors have made substantial contributions to the work, including editing and writing assistance, reported in the manuscript. FY, RM, CL, and LZ collaborated in the study conception and design. Cases and blocks were selected from the First Affiliated Hospital of Zhengzhou University by KF and MW. Data collection and analysis were performed by FY. The first draft of the manuscript was written by FY, and all authors commented on previous versions of the manuscript. The entire experimental process was supervised and guided by DY. All authors contributed to the article and approved the submitted version.

## Funding

The present study was supported by grants from the University Scientific and Technological Innovation Team Project of Henan Province (19IRTSTHN002); The Thousand Talents Science and Technology Innovation Leading Talents Subsidy Project of Central Plains (194200510011); Major Scientific Research Projects of Traditional Chinese Medicine in Henan Province (No.20-21ZYZD14); Cultivation of Young and Middle-aged Health Science and Technology Innovation Leading Talents in Henan Province (YXKC2020015).

## Conflict of Interest

The authors declare that the research was conducted in the absence of any commercial or financial relationships that could be construed as a potential conflict of interest.
